# Valorisation of spent tire rubber as carbon adsorbents for Pb(II) and W(VI) in the framework of a Circular Economy

**DOI:** 10.1007/s11356-023-27689-5

**Published:** 2023-05-20

**Authors:** Maria Bernardo, Nuno Lapa, Filomena Pinto, Miguel Nogueira, Inês Matos, Márcia Ventura, Ana Maria Ferraria, Ana Maria Botelho do Rego, Isabel Maria Fonseca

**Affiliations:** 1 LAQV/REQUIMTE, Departamento de Química, Faculdade de Ciências E Tecnologia, Universidade Nova de Lisboa, 2829-516 Caparica, Portugal; 2 Laboratório Nacional de Energia E Geologia (LNEG), Unidade de Bioenergia (UB), Estrada Do Paço Do Lumiar, Ed. J, 1649-038 Lisbon, Portugal; 3 Departamento de Engenharia Química, BSIRG, IBB - Institute for Bioengineering and Biosciences, Instituto Superior Técnico, Universidade de Lisboa, 1049-001 Lisbon, Portugal; 4 Associate Laboratory i4HB—Institute for Health and Bioeconomy at Instituto Superior Técnico, Universidade de Lisboa, Av. Rovisco Pais, 1049-001 Lisbon, Portugal

**Keywords:** Spent Tire Rubber, Chars, Adsorption, Lead, Tungsten

## Abstract

Spent tire rubber-derived chars and their corresponding H_3_PO_4_ and CO_2_-activated chars were used as adsorbents in the recovery of Pb(II) ion and (W(VI)) oxyanion from synthetic solutions. The developed chars (both raw and activated) were thoroughly characterized to have insight about their textural and surface chemistry properties. H_3_PO_4_-activated chars presented lower surface areas than the raw chars and an acidic surface chemistry which affected the performance of these samples as they showed the lowest removals of the metallic ions. On the other hand, CO_2_-activated chars presented increased surface areas and increased mineral content compared to the raw chars, having presented higher uptake capacities for both Pb(II) (103–116 mg/g) and W(VI) (27–31 mg/g) ions. Cation exchange with Ca, Mg and Zn ions was appointed as a mechanism for Pb removal, as well as surface precipitation in the form of hydrocerussite (Pb_3_(CO_3_)_2_(OH)_2_). W(VI) adsorption might have been ruled by strong electrostatic attractions between the negatively charged tungstate species and the highly positively charged carbons’ surface.

The results shown in this work allow concluding that the valorisation of spent tire rubber through pyrolysis and the subsequent activation of the obtained chars is an alternative and a feasible option to generate adsorbent materials with a high uptake capacity of critical metallic elements.

**Supplementary Information:**

The online version contains supplementary material available at 10.1007/s11356-023-27689-5.

## Introduction

The management of spent tires (ST) represents a challenge worldwide. Valorpneu (the Portuguese Management Company of the Integrated System for Used Tires) reported that in 2021 around 74,000 tons of ST were generated in Portugal (Valorpneu 2022), while in the European Union that value reached 3.55 million tons in 2019 (ETRMA 2021). In Europe, the rubber of 55% of the collected ST was recycled (shredded, granulated, or powdered to children’s playgrounds, sports surfaces, asphalt and concrete incorporation, among other uses), mainly through mechanical recycling (ETRMA 2021). However, the problem remains at the end-of-life (EoL) of these rubber products: how to manage the spent rubber and how to separate it from the other components of the composite materials? In 2019, of the total ST generated in Europe, 40% was sent to energetic valorisation (combustion), and 5% was classified as unknown/landfill destination (ETRMA 2021), despite the European Landfill Directive (1991/31/EC) forbids the landfilling of EoL tires (EoLT) since 2006. Energetic valorisation includes the use of ST as a supplemental fuel in power plants and cement kilns by co-incineration, however, this implies the destruction of material that could be converted into valuable raw materials or fuel and the emission of greenhouse gases, namely CO_2_. Only a residual amount (< 1%) was chemically recycled. Therefore, recycling industries are focused on increasing the value of secondary raw materials derived from tires through innovative and sustainable pathways based on the concept of Circular Economy (Araujo-Morera et al. 2021).

Pyrolysis, also known as thermochemical recycling, has demonstrated to be a viable route for spent tire rubber valorisation, as it effectively converts the rubber into high added-value products such as oils, gases, and carbon-rich solids (recycled carbon black or chars), thus consisting in a recovery process of energy and materials (dos Santos et al. 2020). Depending on the pyrolysis conditions, the carbonaceous char can be produced with a yield of around 30–40% wt, mainly composed of the initial carbon black, inorganic compounds (like Zn, Si, and Ca) and other carbon-based solids generated by re-polymerization of butadiene rubber (BR) and styrene-butadiene rubber (SBR) (Hita et al. 2016). The char can be reused as recycled carbon black filler for rubber materials, however, it has to suffer extensive purification processes for surface modification, elimination of the carbonaceous deposits, ash, sulphur content, and odour to comply with the required high standards of rubber industry, which significantly hinders its reuse in these industries (Xu et al. 2020). Common research on the tire-derived char has been its direct use or its use as a precursor of high-value activated carbons (Saleh and Gupta 2014; Jones et al. 2021), which opens the window for a wide range of applications in different industries. Among the different applications of the tire rubber-derived carbons, their unique properties have made them highly efficient as adsorbents of a wide range of compounds (Kuśmierek et al. 2021b, a).

On the other hand, wastes of electric and electronic equipment (WEEE) and waste batteries and accumulators (WBA), constitute two types of rising problems in high-tech societies (Dehghani-Sanij et al. 2019; Shittu et al. 2021). Currently, around 10 million tonnes per year of WEEE and WBA are generated in European countries (European Commission 2021a, b). Therefore, increasing the collection, treatment, and valorisation of these kinds of wastes is an urgent need. Nevertheless, not all these wastes are properly collected and recycled at the EoL, increasing the risk of environmental contamination by hazardous substances. Additionally, the loss of WEEE and WBA represents a loss of resources with high economic value. The recovery of specific elements from WEEE and WBA, due to their economic importance, supply risk, and hazardousness, is a priority in the Circular Economy action plan and an environmental emerging issue.

Among the different treatment steps of WEEE and WBA (Kaya 2016), chemical decomposition by leaching or chemical treatment for later recovery of target metals is critical. This recovery can be accomplished through the adsorption of the metals of interest from the leachates by using efficient adsorbents.

In this context, the present work aims to study the valorisation of ST rubber through pyrolysis and activation/functionalization into high added-value carbon materials to be subsequently used as adsorbents in the recovery of lead ions (Pb(II)) and tungsten oxyanions (W(VI)) from synthetic solutions. Lead is a common element used in batteries and other electric and electronic equipment (Li et al. 2016; Hao et al. 2020), with demonstrated toxicity to the environmental ecosystems and human health (Ravipati et al. 2021). Tungsten is considered by the European Commission as a critical raw material, due to its high economic importance and supply risk to Europe (European Commission 2020).

This study provides new knowledge about alternative recycling pathways of ST rubber through pyrolysis process into carbon products whose properties will be improved by using different strategies to efficiently recover both Pb cations and W anions from aqueous solutions. ST rubbers from different origins will be used to assess the influence in the composition of the rubbers and in the corresponding carbon materials. An in-depth study of the developed carbon products will provide insight on their properties and on the associated removal mechanisms of both ions.

## Materials and methods

### *Samples of Spent Tire Rubber*

Two types of ST rubber were used as precursors of carbon materials: Sample A, which is a cryogenic recycled rubber from tires of light vehicles (particle size: 0.18—0.60 mm); Sample B, which is a mechanically recycled rubber obtained from a mixture of tires of light and heavy-duty vehicles (particle size: 0.6—0.8 mm). Both rubber samples were submitted to the following characterizations:

i) Elemental analysis (C, H, N, and S) (Thermo Finnigan-CE Instruments Flash EA 1112 CHNS analyzer);
ii) Thermogravimetric analysis (TGA) (30—900 °C, 5 °C/min, argon flow (Setaram Labsys EVO);
iii) Ash content (750 ºC, 5 °C/min, for 3 h in a muffle furnace);
iv) Mineral content, based on the EN 15,290 standard. The samples were acid-digested (3 mL H_2_O_2_ 30% v/v + 8 mL HNO_3_ 65% v/v + 2 mL HF 40% v/v) in a microwave station (Milestone Ethos 1600 Microwave Labstation) and neutralized (20 mL H_3_BO_3_ 4% w/v). The acidic solutions were analyzed by inductively coupled plasma–atomic emission spectroscopy (ICP-AES) (Horiba Jobin–Yvon) for the quantification of several chemical elements.

### Pyrolysis and Chars

The pyrolysis assays were carried out in a stirred batch reactor (Parr Instruments, Hastelloy C276) which was purged and pressurized to 0.6 MPa with nitrogen (N_2_). A heating rate of 5 °C/min was applied until the desired reaction temperature of 405 °C had been reached, which was then held for 30 min. After cooling to room temperature, the resulting chars were submitted to sequential extractions with hexane and acetone to remove the pyrolysis oil and tars soaked in the chars. Finally, the chars were washed with water. The chars obtained from rubber samples A and B were coded as CA and CB, respectively, and submitted to the following characterizations:

i) Elemental analysis, TGA, and mineral content as described above;
ii) Ash content according to ASTM D1762 standard (750 ºC for 6 h);
iii) pHpzc was determined by preparing solutions of 0.1 M NaCl with initial pH values between 2.00 and 12.00. The pH correction was performed with solutions of NaOH and HCl with concentrations between 0.01 and 1 M. A mass of 0.1 g of char was added to 20 mL of each 0.1 M NaCl solution. The mixtures were shaken in a roller-table device for 24 h. pHpzc corresponds to the plateau of the curve pH_final_ *vs* pH_initial_;
iv) X-ray powder diffraction (XRPD), in which the diffractograms were obtained by using a benchtop X-ray diffractometer (RIGAKU, model MiniFlex II), with a Cu X-ray tube (30 kV/15 mA) by continuous scanning from 15° to 80° (2Ɵ) with a step size of 0.01° (2Ɵ) and a scan speed of 2°/min. Tentative identification of XRD peaks by matching with ICDD and COD databases of XRD software was performed;
v) Fourier Transform Infrared Spectroscopy (FTIR) by the KBr disk method (Perkin-Elmer-Spectrum 1000 Spectrometer) in the 4000–400 cm^−1^ range under a resolution of 1 cm^−1^;
vi) N_2_ adsorption–desorption isotherms at -196 ºC obtained in an ASAP 2010 Micromeritics equipment. The adsorption data were used to calculate the apparent surface area (A_BET_) through the BET equation. The total pore volume (V_total_) was determined by the amount of N_2_ adsorbed at the relative pressure p/p_0_ = 0.95. The micropore volume (V_micro_) was evaluated by the t-plot method, and the mesopore volume (V_meso_) was determined by the difference between V_total_ and V_micro_. The samples were previously outgassed overnight, under vacuum pressure, at 150 °C.
vii) Scanning Electron Microscopy with Energy Dispersive Spectroscopy (SEM–EDS) – The morphology and elemental composition of carbon samples were obtained by using a JEOL 7001F analytical FEG-SEM with energy dispersive X-ray spectrometer light element detector.
viii) Confocal Raman spectrophotometer (Witec α 300 RAS) using a laser with a wavelength of 532 nm and 1 mW of power was also used for complementary composition characterization.
ix) X-ray Photoelectron Spectroscopy (XPS) was used to characterize the chars chemical compositions. A XSAM800 non-monochromatic dual anode spectrometer from Kratos was used. Spectra were obtained using the Mg Kα X–ray source (1253.6 eV). Given the large charge accumulation (charge shift larger than 1 eV) detected in the non-activated chars (samples CA and CB), experimental binding energies (BE) were corrected from this charge shift assuming that the contents in graphitic carbon are very low and, therefore, the main component in C 1 s was assigned to sp3 carbon atoms, set to 285 eV. Further details on the operation conditions, spectra acquisition and data treatment were described elsewhere (Salomé et al. 2016). For quantification purposes, the sensitivity factors (SF) used were furnished by the library from software Vision 2 for Windows, Version 2.2.9 from KRATOS: 0.278 for C 1 s, 0.78 for O 1 s, 0.668 for S 2p, 0.328 for Si 2p, and 3.726 for Zn 2p3/2.

### Activated chars

CA and CB chars were then activated with CO_2_ (physical activation) and H_3_PO_4_ (chemical activation). Physical activation was performed in a quartz reactor placed in a custom-made electric vertical tube furnace at 800 °C (heating rate of 10 °C/min) for 6 h, under a CO_2_ flow of 100 mL/min. The activated chars obtained were coded as CA-CO2 and CB-CO2.

In the chemical activation process, the chars were first impregnated with H_3_PO_4_ under a mass ratio of 1:1, at 50 °C, for 5 h, and then dried at 130 °C; the impregnated chars were placed in a quartz reactor inside the furnace and activated at 500 °C (heating rate of 5 °C/min), for 2 h, under an N_2_ flow of 150 mL/min. Finally, the obtained carbons were thoroughly washed with deionized water until stable pH. The samples obtained from the chemical activation of chars A and B were coded as CA-H3PO4 and CB-H3PO4, respectively.

The activated chars were characterized for elemental analysis, ash content, TGA, XRPD, FTIR, pH_PZC_, N_2_ adsorption–desorption isotherms at 77 K, Raman spectroscopy, SEM–EDS and XPS as described above for raw chars. XPS spectra of activated chars were corrected from the charge shift using as reference the BE of a mixture of C 1 s sp3 and sp2 set to 284.7 eV (Beamson and Briggs 1992). The experimental BE in the activated samples were systematically below 285 eV (~ 284.9 eV) and no additional charge compensation (e.g. electron flood gun) was used. The same SF as for non-activated chars were considered in the quantitative analysis plus that of P 2p equal to 0.723.

### Adsorption assays

A synthetic solution with an initial W(VI) concentration of 100 mg/L was prepared by diluting a standard Ammonium Tungstate (NH_4_)_2_WO_4_ solution of 1000 mg/L (Scharlau) with deionized water. Also, a synthetic Pb(II) solution with an initial concentration of 100 mg/L was prepared through the dissolution of Pb(NO_3_)_2_ salt (Merck) with deionized water.

Batch adsorption experiments were performed in 20 mL vials, at room temperature, under constant agitation in a multi-point stirrer. After each adsorption assay, the samples were filtered through vacuum by using 0.22 µm MCE membranes. The filtrates were analyzed by ICP-AES for W(VI) and Pb(II) quantification. Duplicates were prepared for each assay.

The effect of pH on metal ion removal was evaluated for a pH range of 2–5 for Pb(II) and 2–7 for W(VI). These assays were performed with an adsorbent mass of 30 mg and 10 mL of solution with an initial metal concentration of 100 mg/L, and agitation for 24 h. The effect of contact time on the uptake capacity (kinetic study) of adsorbents was performed up to 72 h, at pH 5 for Pb(II) and pH 2 for W(VI), with an adsorbent mass of 10 mg and 10 mL of solution, with an initial metal concentration of 100 mg/L. The influence of different initial concentrations (equilibrium studies) was performed at a contact time of 48 h, an adsorbent mass of 10 mg, and 10 mL of solution with an initial metal concentration ranging from 20 to 200 mg/L for both metal ions.

W(VI) and Pb(II) removal efficiencies, R (%), and adsorbent uptake capacity, qt (mg/g), were calculated by Eqs. 1 and 2, respectively:

1 $$R (\%)=\frac{\left({C}_{0}-{C}_{f}\right)\times 100}{{C}_{0}}$$

2 $${q}_{t}= \frac{\left({C}_{0 }-{C}_{f}\right)\times V}{m}$$

where *C*_*0*_ and *C*_*f*_ are the initial and final concentrations (mg/L) of the metal ions, respectively, *V* (L) is the volume of solution, and *m* (g) is the adsorbent mass.

## Results and discussion

### Samples characterization

The yields of pyrolysis chars CA and CB after solvents extraction were 47.1% wt. and 51.8% wt, respectively. This result shows that a substantial amount of pyrolysis oil and tars rich in aromatic, cyclic, and aliphatic hydrocarbons can be removed and recovered from the chars (Bernardo et al. 2012; Williams 2013).

The results from the characterizations performed on the rubber samples, chars, and activated chars are presented in Table 1. It can be observed that both rubbers have similar CHNS compositions, as well as ash content. The pyrolysis chars (CA and CB) presented a higher ash content than the rubbers, resulting from the concentration effect of the pyrolysis process. Also, the sulfur content increased in the produced chars, indicating that this element has a low volatilization degree at the pyrolysis temperature used in the present work being retained in the carbon matrix (Martínez et al. 2013). These chars presented neutral pHpzc values and their composition is in agreement with previous studies of rubber tire-derived chars (Cardona et al. 2018; Yu et al. 2020). The carbons resulting from H_3_PO_4_ activation presented a much smaller sulfur content compared to the raw chars, as well as low pHpzc. Acidic activation and the extensive H_2_O washing of the produced carbons may have promoted the removal of sulfide compounds. On the other hand, the low pHpzc indicates the introduction of acidic groups on the carbons’ matrix. Nevertheless, the ash content increased despite the intensive washing of the resulting carbons; non-soluble inorganic matter was retained on the resulting carbons. The carbons from CO_2_ activation presented increased basicity, probably due to a mineral concentration effect, which is confirmed by the higher ash content. In this case, the carbons’ sulfur content was not reduced.

**Table 1:** Elemental analysis, ash content, and pH_PZC_ of rubber and carbon samples (as received basis)

	C (%)	H (%)	N (%)	S (%)	Ashes (%)	pHpzc
Rubber A	79.20	7.07	0.40	1.64	9.50	n.d
Rubber B	83.39	7.60	0.40	2.04	8.70	n.d
CA	71.33	0.71	0.28	2.51	21.4	7.4
CB	79.06	0.86	0.33	3.94	13.9	6.7
CA-H3PO4	69.00	0.52	0.20	0.42	23.8	3.0
CB-H3PO4	70.16	0.48	0.24	0.44	21.3	2.8
CA-CO2	70.26	0.16	0.23	2.90	27.3	8.5
CB-CO2	76.26	0.14	0.29	3.70	17.5	7.8

n.d. – not determined.

The N_2_ adsorption–desorption isotherms of the carbon samples as well as the textural parameters obtained from the isotherms are presented in Figure S1. The chars presented both low surface areas and total pore volumes, which are typical for this type of material (Cardona et al. 2018; Yu et al. 2020). After the activation with H_3_PO_4_, there was a slight decrease in the surface area, possibly due to pore blocking with functional groups or some destruction of the porosity. The activation with CO_2_ allowed a small increase in the surface area due to the gasification reactions, but the burn-off (carbon gasification conversion) was low (12.5–15.0%).

The isotherms are of type IV(a) according to the IUPAC classification (Thommes et al. 2015), typical of mesoporous materials.

The hysteresis loop of type H3 is associated with non-rigid aggregates of plate-like particles with slit-shaped pores and/or macropores that are not completely filled with pore condensate. However, for very thin powder samples, as is the case of these tire-derived carbons, the N_2_ isotherms show a sharp rise in the adsorbed amount at high relative pressures (p/p_0_ > 0.9) associated with interparticle porosity rather than bulk porosity (Sotomayor et al. 2018). These N_2_ isotherms are quite similar to the ones obtained by other authors concerning tire-derived chars (Wang et al. 2019; Betancur et al. 2020; Pan et al. 2021). The micropore size distributions obtained from the DFT adsorption model for carbon slit-shaped pores (Figures S2 and S3) reveal that the activation of chars increased the volume of narrow micropores, being especially visible for the CO_2_-activated chars. In contrast, H_3_PO_4_ destroyed the existent microporosity in char CB (Figure S3).

The mineral content of the rubber samples and raw chars was determined, and the results are presented in Table 2.

**Table 2:** Mineral content of rubber and raw char samples ($\overline{X }\pm \sigma , n=2$)

Concentration (mg/g)	Rubber A	Rubber B	CA	CB
Zn	29.2 ± 0.19	38.6 ± 5.37	69.6 ± 1.75	93.5 ± 0.79
Ca	13.0 ± 3.9	6.38 ± 0.18	21.9 ± 0.52	11.9 ± 0.82
Fe	2.18 ± 0.55	4.25 ± 0.27	4.96 ± 0.09	8.75 ± 0.15
Mg	0.815 ± 0.239	0.870 ± 0.105	1.79 ± 0.09	1.74 ± 0.01
Cu	0.473 ± 0.049	1.02 ± 0.17	0.318 ± 0.014	1.63 ± 0.03
Pb	0.081 ± 0.027	0.043 ± 0.001	0.118 ± 0.003	0.112 ± 0.000
Ti	0.067 ± 0.009	< 0.004	< 0.004	< 0.004
Mn	0.029 ± 0.008	0.025 ± 0.002	0.052 ± 0.006	0.059 ± 0.006
Ba	0.202 ± 0.02	< 4 × 10^–5^	< 4 × 10^–5^	< 4 × 10^–5^
Cr	0.002 ± 0.002	0.006 ± 0.003	0.008 ± 0.000	0.010 ± 0.003
Ni	0.004 ± 0.000	0.004 ± 0.001	0.012 ± 0.000	0.016 ± 0.000
Mo	< 4 × 10^–4^	< 4 × 10^–4^	0.036 ± 0.000	0.120 ± 0.006

$\overline{X }\pm \sigma$: average ± standard deviation; $n$: number of samples analyzed.

Rubber B is richer in Zn and Fe; whilst rubber A presents more Ca, highlighting the influence of the different spent rubber sources (tires of light vehicles and from heavy-duty vehicles) in the mineral composition of chars.

The chemical elements Al, Cd, K, Na, Se, Si, and Sn were not detected in the samples. Generally, all the elements presented higher concentrations in the resulting chars compared to the rubbers, due to the concentration effect of pyrolysis. The concentrations of Zn stand out since ZnO is used in the vulcanization process of tire rubber (Coran 2013). Ca also showed high concentrations followed by Fe, Mg, and Cu.

**Fig. 1:**
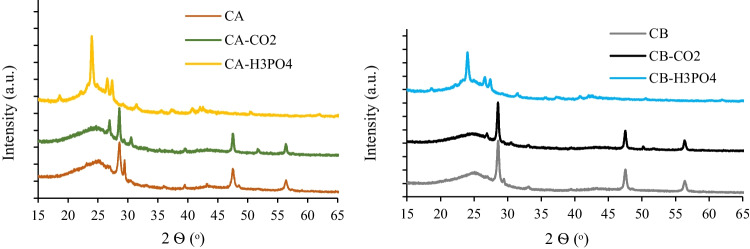
XRDP pattern of carbon samples (left: char and activated chars from rubber **A**; right: char and activated chars from rubber **B**)

XRD analysis allowed identifying the main crystalline mineral phases in carbon samples (Fig. 1).

All the samples presented a broad diffraction band between 15 − 30° (2Ɵ) associated with an amorphous carbon structure. The raw chars presented typical diffractograms of spent tire-derived chars (Smith et al. 2016; Seng-eiad and Jitkarnka 2016), where peaks attributed to ZnS in the form of sphalerite (β-ZnS) and wurtzite (α-ZnS), formed due to the reaction of S with the ZnO present in the tire rubber during pyrolysis, can be identified (Lin et al. 2008; López et al. 2013). The chars activated with CO_2_ presented a similar XRD pattern compared to the raw chars, although some peaks become sharped, due to the phase transition of sphalerite to wurtzite (Li et al. 2020). The samples activated with H_3_PO_4_ present a distinctive diffractogram with the disappearance of zinc-derived peaks and the appearance of new peaks mainly associated with the silicophosphate (SiP_2_O_7_) phase due to the reaction of amorphous silica with H_3_PO_4_ (Khabbouchi et al. 2018). Silicon was not detected in the mineral content of the raw chars (Table 2), probably because it was not solubilized in the acidic digestion. The activation promoted the concentration of this element that reacted with the acid, producing a crystalline compound. In fact, silica (SiO_2_) is commonly used as a reinforcement filler in tires (Bockstal et al. 2019) and, as it will be demonstrated with further analyses, silicon is actually present in the chars.

**Fig. 2:**
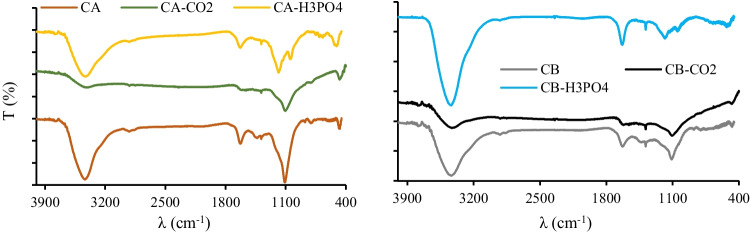
FTIR spectra of carbon samples (left: char and activated chars from rubber **A**; right: char and activated chars from rubber **B**)

Figure 2 presents the FTIR spectra of carbon samples.

Overall, the spectra are very similar with common bands at 3430 cm^−1^ (stretching vibration of hydroxyl groups O–H mainly attributed to water), 2920 cm^−1^, and 1384 cm^−1^ (C-H stretching vibrations of methyl and methylene groups), 1630 cm^−1^ (C = C or C = O stretch vibration), and in the range 900–750 cm^−1^ (aromatic C-H out-of-plane bend) (Coates 2006; Acevedo and Barriocanal 2015; Seng-eiad and Jitkarnka 2016). Also, all samples presented a strong band at 1100 cm^−1^ that can be assigned to silicon-oxy compounds, Si–O–C or Si–O–Si (Coates 2006), thus confirming the presence of silica. Samples derived from H_3_PO_4_ activation presented a new and distinctive band at 1035 cm^−1^ and a new group of small bands between 750–650 cm^−1^ that can be associated with P-O-Si, Si–O-Si, or P-O-P vibrations (Khabbouchi et al. 2020).

TGA analysis of rubber and carbon samples is shown in Figure S4. Both rubbers A and B presented the main decomposition at a temperature range between 260–470 °C corresponding to a mass loss of around 60%, remaining 30 to 40% (w/w) of char product at 900 °C. The raw chars as well as the chars activated with CO_2_ showed high thermal stability with a total mass loss below 10% (w/w). The activated chars with H_3_PO_4_ also presented thermal stability up to 750 ºC. Above this temperature, they lose weight significantly up to 900 °C which could be due to the decomposition of phosphorous compounds (Chen et al. 2019).

**Fig. 3:**
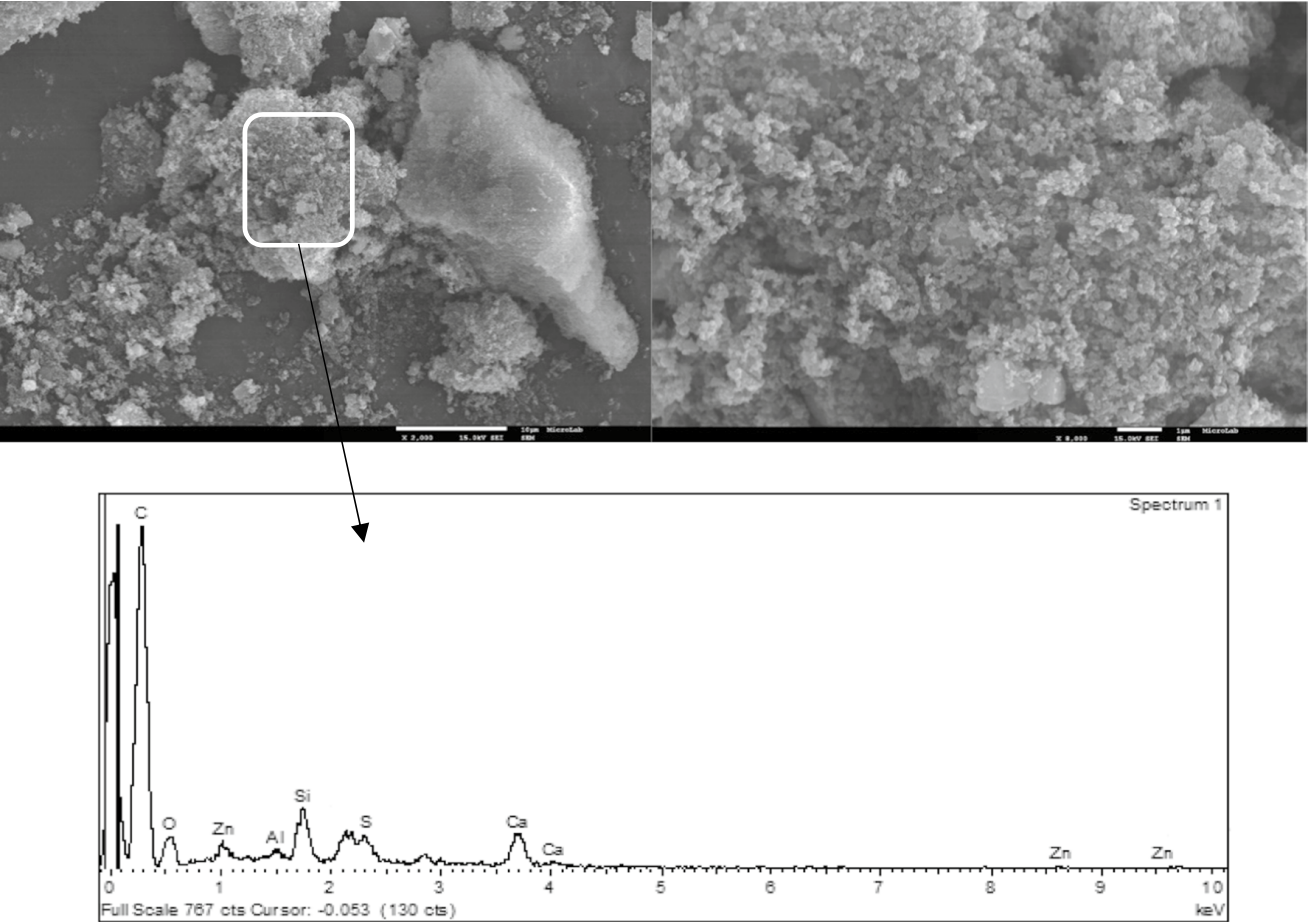
SEM images of CA char particles with 2000 × (left) and 8000 × (right) magnification and the corresponding EDS spectrum

**Fig. 4:**
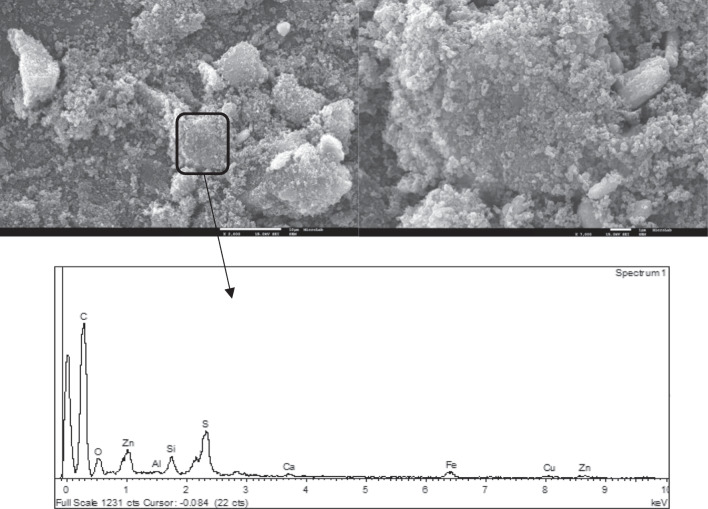
SEM images of CB char particles with 2000 × (left) and 7000 × (right) magnification and the corresponding EDS spectrum

Figures 3 and 4 present the SEM images of char particles and the EDS spectra.

The surface morphology of the particles is typical of that of tire rubber-derived chars where rough spherical carbon black aggregates can be observed covered with the tarry by-products of pyrolysis, and some mineral clusters can also be observed (Moulin et al. 2017; Martínez et al. 2019; Xu et al. 2021). Some authors suggested a core–shell structure for tire rubber-derived char particles: in the pyrolysis process, the spherical carbon black particles (used as reinforcing filler in the tire rubber) act as nuclei seed (core) promoting the growth of carbonaceous structures through secondary reactions around them which constitutes then the shell (Cardona et al. 2018; Yu et al. 2020). The aggregates present an irregular distribution of particle sizes up to 100–500 nm constituting agglomerates (due to van der Waals attraction forces between particles but also due to the binder effect of carbonaceous deposits), with sizes ranging from 1 to 10 µm (Cardona et al. 2018).

The EDS spectra of the selected char particles show a predominance of the carbon element but the presence of other elements such as O, S, Zn, Al, Ca, Fe, and Cu were confirmed, as well as the presence of Si.

Distinct surface morphology can be seen in the obtained activated carbons. The SEM images of CO_2_-activated carbons (Figures S5 and S6) revealed a decrease in the flake-like structure of the particles, at least for the same magnification. A smoother surface can be a result of the gasification of the carbonaceous deposits formed around the carbon black particles. EDS spectra of the particles of these activated carbons are quite similar to the spectra of the raw chars indicating that the mineral composition did not change significantly. On the contrary, the particles of activated chars with H_3_PO_4_ presented EDS spectra with new peaks (Figures S7 and S8), namely peaks assigned to P and Si elements, corroborating the hypothesis that silicophosphate compounds were formed due to the reaction of amorphous silica with H_3_PO_4_. The SEM images of these carbons’ particles revealed a rough surface with much more mineral clusters coating the surface.

The most abundant elements were also studied in detail by XPS, in particular those quantified in Table 3, which shows the overall quantification.

**Table 3:** XPS atomic concentrations (%) of relevant elements in all carbons samples

	CA	CA-CO2	CA-H3PO4	CB	CB-CO2	CB-H3PO4
C	65.4	68.7	64.7	76.8	79.5	62.2
O	23.8	22.6	29.9	17.4	15.2	32.2
S	0.4	0.4	0.1	0.5	0.9	0.1
Si	9.9	7.5	1.1	4.7	3.3	1.1
Zn	0.5	0.8	0.2	0.6	1.0	0.2
P			4.0			4.3

The most abundant elements are carbon and oxygen, followed by silicon and phosphorus (for H3PO4-activated chars). Sulphur and zinc, in spite of their very low relative amounts, are also perfectly detected after a single acquisition sweep. The presence of other minoritary elements cannot be discarded, in spite of not being detected in the survey spectra (not shown). Figure S9 shows the different XPS regions analysed.

The horizontal double arrow indicates differential charge, which results from different parts of the sample with different conductivities and/or a built-up contact potential at given points of the sample. In both cases, an enlargement of the spectrum is observed.

C 1 s most intense fitted peak, centred at 285 or 284.7 eV, is assigned mainly to sp3 or a mixture of sp3 and sp2 carbon atoms, respectively. The presence of sp2 carbon atoms is also attested by the energy loss regions, due to π-π* excitations, detected in C 1 s roughly between 290 and 296 eV. The peak centred at 286.1 ± 0.1 eV could be assigned to carbon atoms singly bonded to oxygen, however this assignment is not consistently attested by the quantitative analysis. Therefore, this peak is most probably resulting from the intrinsic asymmetry of a C 1 s peak from graphitic carbon. Peaks centred between 287.1 and 288.8 eV are assigned to epoxides, carbonyl groups and carboxylates (typically found at 287.0, 287.9 and 289.0, respectively). At 289.4 ± 0.2 eV, carbon in carboxylic groups is detected in CA, CA-CO2, CB-CO2 and CB-H3PO4 (Beamson and Briggs 1992). The fraction of -COOH decreases in CA chars after CO_2_ activation (CCOOH/CTotal = 0.04 and 0.02, in CA and CA-CO2, respectively), which is compatible with the increased basicity described above. However, regarding the very low intensity of this spectral feature and the differential charge detected in CB-CO2, no further conclusions can be drawn from the remaining samples analyses. Still, one can notice that both CA-CO2 and CB-CO2 have a larger relative amount of Zn than the other samples (Table 3), which is in accordance with the percentage of ashes measured (Table 1).

The oxigenated carbon functional groups, detected in C 1 s, can also be found in O 1 s, but with less resolution (because chemical shifts in O 1 s are lower than in C 1 s). The peaks assigned to these organic functional groups are mixed with the peaks from the inorganic species, such as silica, phosphates or sulfates.

Silicon is clearly detected by XPS in all samples, mainly in the form of silica. Si 2p is a doublet with a spin–orbit split of 0.61 eV. Si 2p3/2 of silica is usually found between 103 and 104 eV (Naumkin et al. 2012). However, in non-activated and CO2-activated chars, Si 2p3/2 BE is higher than 104 eV. This over (unexpected) energy shift in Si 2p, is also observed in O 1 s: peaks fitted over ~ 534 eV are most probably from oxygen atoms in charged SiO2. This effect may be due to silica particles being loosely distributed within the chars material, i.e. not tightly bonded to the carbon’s matrix, and for that reason conducting differently than the matrix. These attributions are confirmed by the quantitative analysis, which shows OBE > 534 eV/Si ≥ 2 (may also include surface Si–OH); oxygen in uncharged SiO2 is expected at 532.5 – 533.2 eV (Naumkin et al. 2012).

For the H_3_PO_4_ activated chars, the phase detected by XRD, SiP_2_O_7_, although not directly identified from the XPS spectra, is not contradicted by XPS quantitative analysis, which reveals atomic ratios P/Si and O (bonded to P and/or Si)/(P or Si) larger than those predicted for SiP_2_O_7_. Therefore, at least part of the silicon and of the phosphorus detected may be in this silicophosphate structure. Furthermore, a more electronegative silicon neighbourhood is expected to increase its chemical shift, hence increasing its BE. However, the Si 2p, for these particular chars (H3PO4-activated) is detected at lower BE than in the other samples, which is, in fact, compatible with the existence of more conductive silicophosphates structures: the electrical conductivity of SiP_2_O_7_ is 2.96 × 10^−6^ Scm^−1^, while that of SiO_2_ is ~ 10–12 Scm^−1^ (Khabbouchi et al. 2019).

The main component of the P 2p doublet (spin–orbit separation = 0.87 eV), P 2p3/2, centred at 134.5 ± 0.1 eV is assigned to PO_4_^3−^ (Naumkin et al. 2012). The oxygen peak from phosphate groups is centred at 533.1 ± 0.2 eV, as attested by the atomic ratio O533.1 eV/P ≥ 4.0 (O533.1 eV can also include some O-C).

XPS also shows that the lowest relative amount of sulfur is obtained for the H_3_PO_4_-activated chars (Table 3), following the results from Table 1. All samples show a S 2p doublet peak, with S 2p3/2 centred at 163.9 ± 0.5 eV (doublet separation = 1.2 eV), assigned to S^2−^ in ZnS. The corresponding Zn 2p3/2 peak is centred at 1022.4 ± 0.2 eV (Naumkin et al. 2012). In CB and CB-CO2, a second S 2p doublet, with S 2p3/2 centred at 169.6 ± 0.1 eV is detected (Other peaks in CB-CO2 S 2p region are due to a differential charge effect). This doublet is attributed to SO_4_^2−^, most probably from ZnSO_4_ (Naumkin et al. 2012). In these samples, the corresponding Zn^2+^ peak is centred at slightly higher BE (1023.3 ± 0.3 eV) than in ZnS, due to the more electronegative neighbourhood. Interestingly, in CA and CA-CO2, where sulfates and phosphates are absent, another peak, centred at 1024.7 ± 0.5 eV, is detected in Zn 2p3/2 regions. This peak is most probably from Zn silicate structures.

**Fig. 5:**
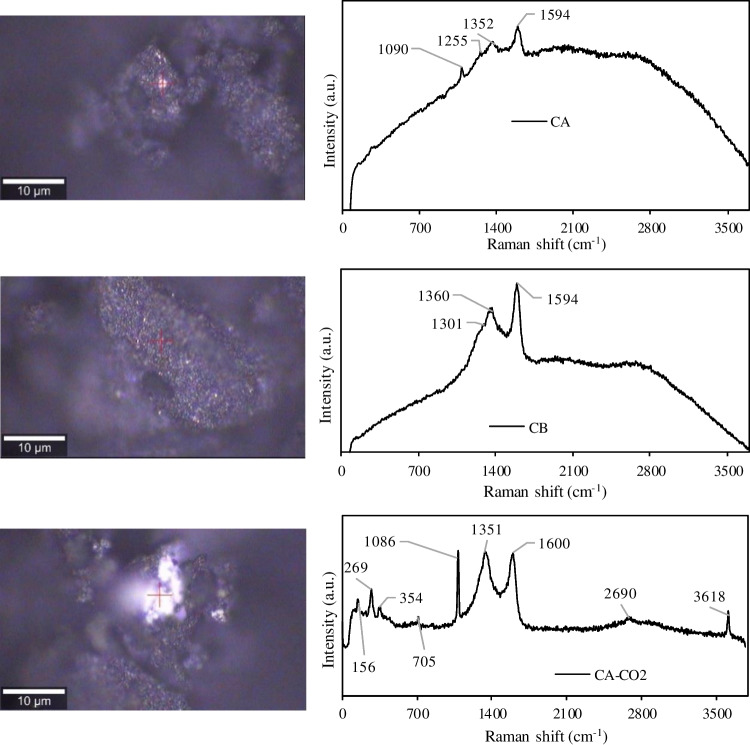

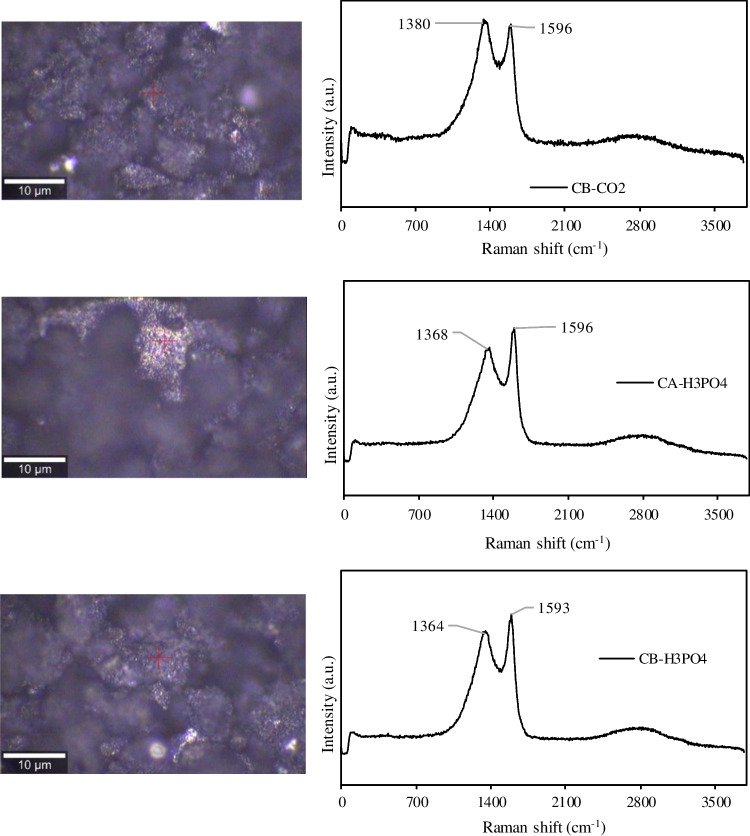
Raman spectra taken from spots on the surfaces of chars and activated chars. The spots are marked in the corresponding microscope images

The Raman study performed on carbon samples provided complementary information about their surface composition and graphitic structure. Figure 5 show the Raman spectra taken from spots on the surfaces of carbon samples. The obtained spectra are typical of amorphous carbon samples with small graphitic planes or microcristallites where two dominant bands can be observed: the D (defects and disorder in the graphitic structure) and G (graphitic structure) bands, centred around 1350 and 1596 cm^−1^, respectively (Ferrari and Robertson 2000). Also, a broad band located between 2500 – 3000 cm^−1^ and associated to the 2D band of graphitic sp^2^ material can be seen in the spectra (Dresselhaus et al. 2010). Similar Raman spectra were previously obtained for tire derived chars attributed mainly to the carbon black present in these carbonaceous samples (Kumar et al. 2018; Sharma et al. 2021). The spectra of CA and CB samples and their resulting H_3_PO_4_ activated chars present G bands of higher intensity than the D bands indicating a more ordered structure. On the contrary, the spectra of the CO_2_ activated chars presented increased intensity for D bands due to the introduction of defects/disorder.

It is possible to identify additional peaks associated to the brighter regions of the chars’ surface, particularly for CA-CO2 sample: peaks at 1086 and 3618 cm^−1^ and several minor peaks between 100 – 400 cm^−1^ are assigned to inorganic compounds (Kumar et al. 2018).

### Adsorption assays

#### Effect of solution pH

The chars and activated chars were then applied as adsorbents of Pb(II) and W(VI) ions from aqueous solutions. The effect of pH on ions removal was evaluated for a pH range of 2–5 for Pb(II) and 2–7 for W(VI). The range of pH selected for Pb is related to the solubility of ion species since Pb starts to precipitate as Pb(OH)_2_ for pH above 7 (Powell et al. 2009). Being an oxyanion, W(VI) adsorption is significantly improved by positively charged adsorbents (Dias et al. 2021), therefore it was not worth it to study pH values above the pH_PZC_ of carbon samples.

**Fig. 6:**
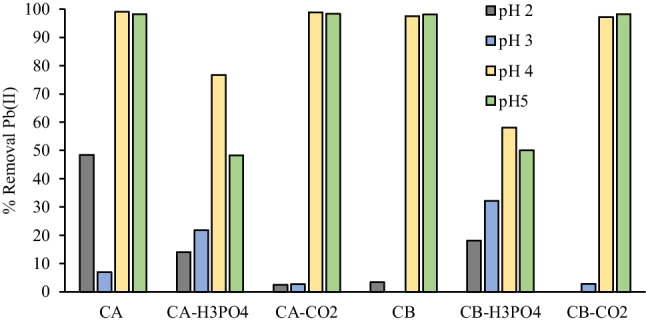
Removal (%) of Pb(II) ions at initial pH ranging from 2–5. Conditions: adsorbent mass = 30 mg; Pb (II) initial concentration = 100 mg/L; solution volume = 10 mL; contact time = 24 h

The higher removals of Pb ions were obtained for initial pH values of 4 and 5 (Fig. 6). The lower removals for acidic conditions should be related to the higher concentration of H^+^ species that competes with Pb(II) ions for the active sites of the carbons.

**Fig. 7:**
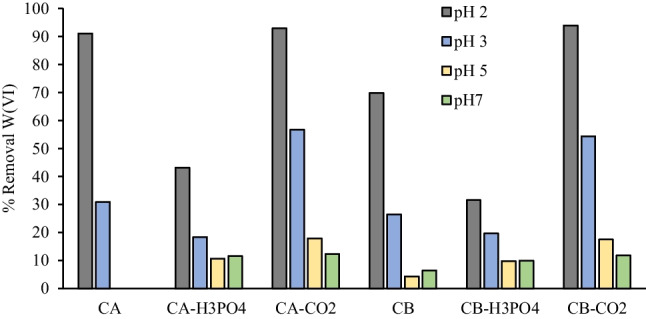
Removal (%) of W(VI) (below) ions at initial pH ranging from 2–7. Conditions: adsorbent mass = 30 mg; W(VI) Initial concentration = 100 mg/L; solution volume = 10 mL; contact time = 24 h

For W(VI) ions, the higher removal rates were achieved for severe acidic conditions (Fig. 7), since the carbons’ surface is strongly protonated with positive charges and can electrostatically attract W(VI) polyoxyanions that can have negative valences (up to –7) under acidic conditions (Smith and Patrick 2000). Higher removals of W(VI) oxyanions at low pH were previously seen with other adsorbents (Sen Tuna and Braida 2014; Wang and Huang 2020; Dias et al. 2021; Wang et al. 2022).

The carbons obtained from H_3_PO_4_ activation presented lower removal percentages for both ions, which can be related to their surface chemistry and textural properties since these samples have lower surface areas (Figure S1). Although these carbons have high mineral content (Table 1), the exchangeability of some of the metallic elements present in the raw chars may have decreased after the activation process. Also, given the low pH_PZC_ values of these carbons, it is expected a negatively charged surface that repels W(VI) anions but attracts Pb(II) ions.

Given the results obtained, it was selected an initial pH of 5 (natural pH) for the next removals assays of Pb(II), and an initial pH of 2 for W(VI). Only the raw chars and activated chars with CO_2_ were selected to be subsequently used.

### Kinetic study

**Fig. 8:**
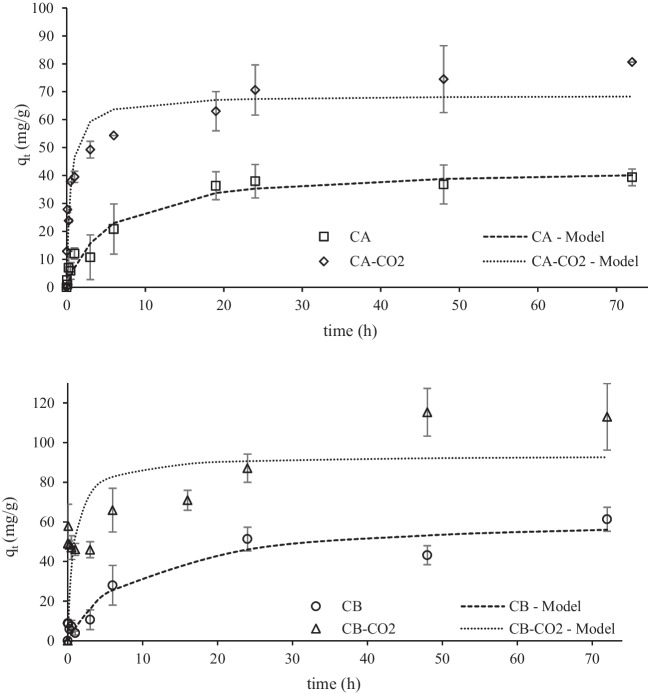
Kinetic data of Pb(II) adsorption adjusted to pseudo 2.^nd^ order kinetic model. Conditions: adsorbent mass = 10 mg; Pb(II) initial concentration = 100 mg/L; solution volume = 10 mL; initial pH = 5

**Fig. 9:**
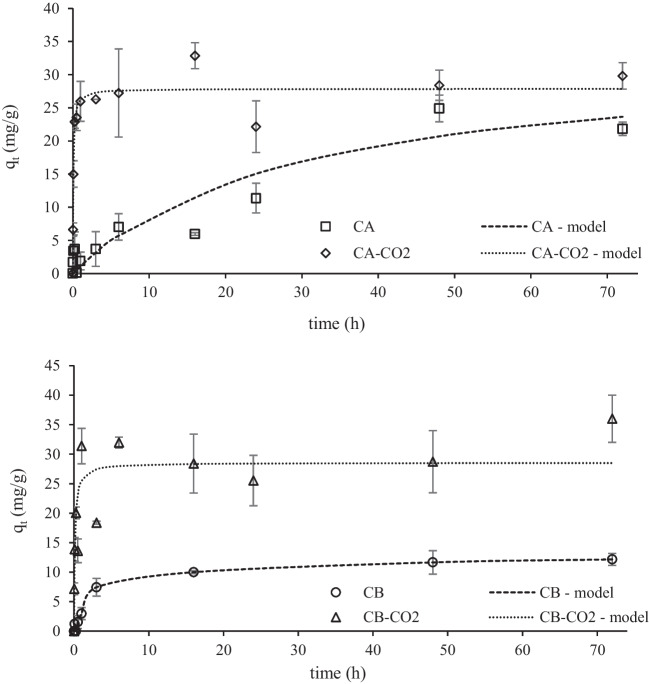
Kinetic data of W(VI) adsorption adjusted to pseudo 2.^nd^ order kinetic model. Conditions: adsorbent mass = 10 mg; W(VI) initial concentration = 100 mg/L; solution volume = 10 mL; initial pH = 2

The results from the kinetic assays of Pb(II) and W(VI) adsorption are shown in Figs. 8 and 9, respectively.

The experimental data was adjusted to both pseudo 1^st^ order and pseudo 2^nd^ order non-linear kinetic models (Lagergren 1898; Ho 2006) by using the minimum of the least-squares method with the SOLVER add-in of MS EXCEL. Overall, the best-fitting model defined by the higher determination coefficient (R^2^) for the kinetic data of both ions for all the adsorbents, was the pseudo 2^nd^ order model. Nevertheless, it should be mentioned that in some cases the data is equally fitted to both models (similar R^2^).

Table 4 shows the kinetic parameters obtained from the modelling of experimental kinetic data to the pseudo 1^st^ and pseudo 2^nd^ order model.

**Table 4:** Kinetic parameters obtained from pseudo 1^st^ order and pseudo 2^nd^ order kinetic modeling of Pb(II) and W(VI) adsorption

		Pb(II)				W(VI)			
		CA	CA-CO2	CB	CB-CO2	CA	CA-CO2	CB	CB-CO2
*Pseudo 1*^*st*^* order*	q_e_ (mg/g)	38.3	64.9	53.3	83.8	25.2	27.0	11.3	27.8
	k_1_ (min^−1^)	0.147	1.53	0.111	1.45	0.038	9.45	0.324	4.13
	R^2^	0.974	0.841	0.936	0.485	0.937	0.918	0.982	0.740
*Pseudo 2*^*nd*^* order*	q_e_ (mg/g)	43.0	68.8	62.9	93.6	33.1	27.9	12.4	28.6
	k_2_ (g/mg.min)	0.004	0.031	0.002	0.013	0.001	0.548	0.03	0.286
	R^2^	0.967	0.897	0.934	0.603	0.932	0.937	0.986	0.776

q_e_—adsorbate uptake at equilibrium; k_1_- pseudo 1^st^ order rate constant; k_2_ – pseudo 2^nd^ order rate constant; R^2^—determination coefficient.

It is possible to conclude that samples from CO_2_ activation presented higher uptake capacities at the equilibrium than the raw chars. In fact, CO_2_ activated chars presented higher surface areas (Figure S1) as well as higher ash content and increased basicity (Table 1). Thus, these samples are richer in cations able to exchange with Pb metallic ions and presented a more positively charged surface able to attract W(VI) anions. Also, the kinetic constants obtained for the assays with the activated chars are the highest, being this fact quite evident for W(VI) adsorption. Usually, mesopore content significantly affects the adsorption kinetics of an adsorbent, but in this case, the mesopore volumes of raw and activated chars are almost the same. An important feature was the observed hydrophobicity of the raw chars pointing out relevant limitations to external mass transfer. This hydrophobic character is related to the spherical carbon black aggregates covered with carbonaceous deposits observed at SEM (Figs. 3 and 4), whereas chars activated with CO_2_ presented cleaner surfaces (Figures S5 and S6) promoting a faster diffusion throughout the boundary layer.

It should be highlighted the weaker adjustment of the kinetic model to the experimental data of the CB-CO2 sample, indicating a complex adsorption mechanism or diffusion limitations. Also, the heterogeneity of the sample revealed by relatively high error bars in the experimental points can explain the weaker adjustment of the theoretical model.

Generally, all the samples achieved adsorption equilibrium for the two ions around 48 h, being this equilibrium time used in the next assays.

### Equilibrium assays – adsorption isotherms

**Fig. 10:**
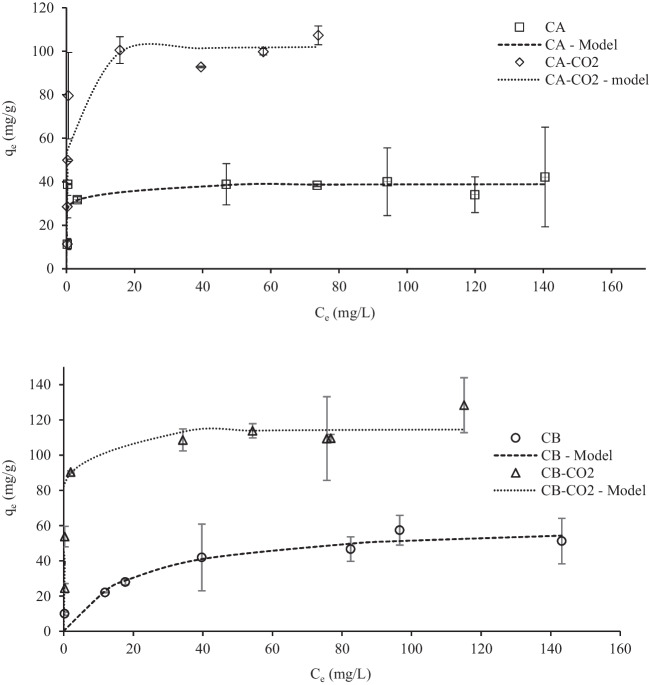
Pb(II) adsorption isotherms adjusted to Langmuir model. Conditions: adsorbent mass = 10 mg; contact time = 48 h; solution volume = 10 mL; initial pH = 5

**Fig. 11:**
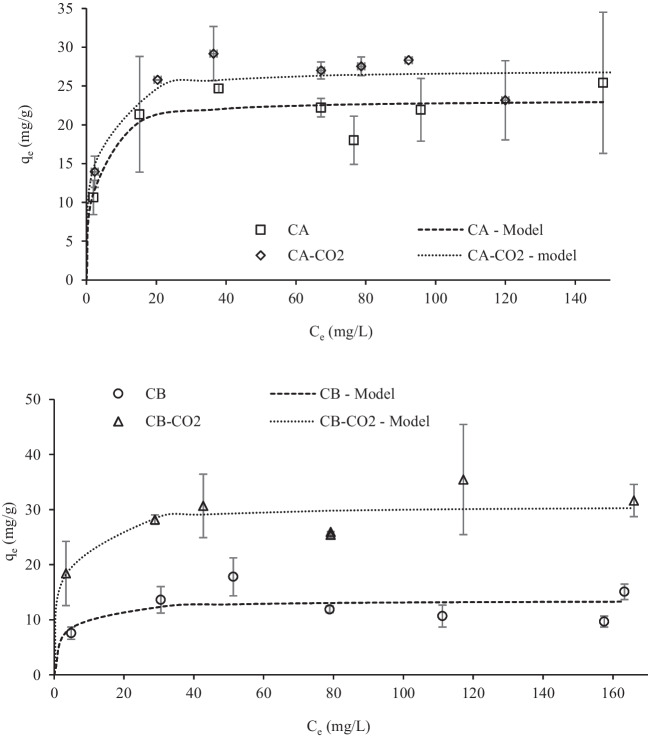
W(VI) adsorption isotherms data adjusted to Langmuir model. Conditions: adsorbent mass = 10 mg; contact time = 48 h; solution volume = 10 mL; initial pH = 2

Pb(II) and W(VI) adsorption isotherms are presented in Figs. 10 and 11, respectively.

Both Langmuir and Freundlich non-linear models were fitted to the experimental data (Freundlich 1907; Langmuir 1917), by also using the minimum of the least-square method with the MS Excel SOLVER tool. The obtained parameters are presented in Table 5 and the theoretical curves obtained with the best fitting model (Langmuir model) are also presented in Figs. 10 and 11.

**Table 5:** Langmuir and Freundlich parameters obtained from modelling of experimental adsorption isotherm data of Pb(II) and W(VI) equilibrium studies

		Pb(II)				W(VI)			
		CA	CA-CO2	CB	CB-CO2	CA	CA-CO2	CB	CB-CO2
*Langmuir*	q_m_ (mg/g)	39.1	103.0	62.1	116.0	23.3	27.1	13.5	30.7
	K_L_ (L/mg)	1.31	2.06	0.049	1.77	0.461	0.508	0.346	0.430
	R^2^	0.978	0.899	0.961	0.955	0.930	0.936	0.749	0.921
*Freundlich*	K_F_^a^	21.3	52.8	12.4	60.5	12.4	16.7	9.10	16.9
	n^b^	7.46	5.71	3.26	6.41	7.35	9.26	13.3	7.81
	R^2^	0.911	0.821	0.959	0.915	0.884	0.855	0.689	0.921

q_m_—monolayer adsorption capacity; K_L_—Langmuir constant; K_F_—Freundlich constant (mg/g)(mg/L)^n^; n—related to the adsorption affinity or surface heterogeneity (dimensionless); R^2^—determination coefficient.

The results confirm the best performance of CO_2_-activated chars, showing the highest maximum uptake capacities of Pb (103–116 mg/g) and W (27–31 mg/g). Also, these carbon samples presented higher Langmuir constants (K_L_) indicating a higher affinity for the ions. Although the Langmuir model is the one that best fitted the experimental data, the Freundlich model also presented a good adjustment to some systems indicating mixed mono and multilayer adsorption.

**Fig. 12:**
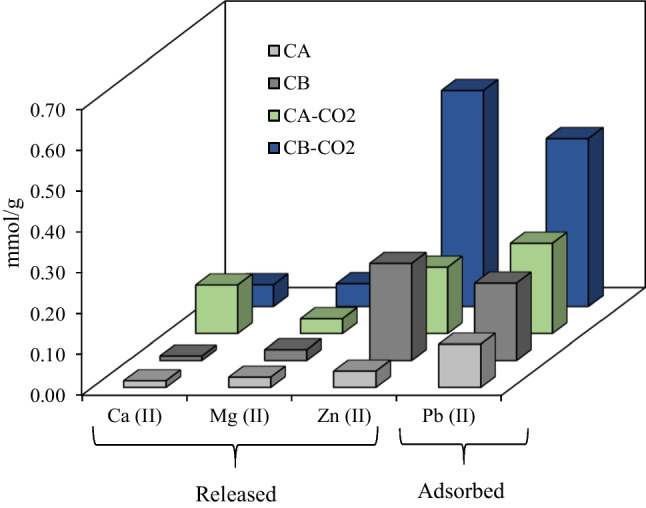
Pb(II) adsorbed and released cations from the chars and activated chars. Conditions: A450 mass = 50 mg; A900 mass = 20 mg; Equilibrium time = 72 h; solution volume = 10 mL

Previous studies with tire-derived chars and activated chars applied as adsorbents of Pb(II) ions did not achieve such high uptake capacities (Table S1). As mentioned above, the higher surface areas of the activated chars (Figure S1) may explain the better performance of these adsorbents. In addition, it was possible to observe that the activated chars presented a much higher volume of narrow micropores (Figures S2 and S3), which can also explain the higher Pb(II) uptakes. Cation exchange has been appointed as a probable mechanism for Pb removal with this type of adsorbents (Quek and Balasubramanian 2009; Chan et al. 2012; Bernardo et al. 2013; Deng et al. 2016), particularly, the exchange with ions that are present in high concentrations on these chars. To confirm this hypothesis, the elements Ca, K, Fe, Mg, and Zn were quantified in the aqueous solutions after the Pb removal assays. These studies were conducted with 10 mg of adsorbent mixed for 48 h with 10 ml of Pb solution with a concentration of 50 mg/L. A blank test with the adsorbent and ultra-pure water was performed. Figure 12 shows the amount of Pb ions adsorbed and the released cations.

Fe and K ions were not detected in the filtrates indicating no releasing from the carbonaceous materials. Ca, Mg and Zn were released, the latter one with significant amounts, supporting the hypothesis of Pb adsorption being driven by cation exchange. The amount of released cations surpass the amount of Pb adsorbed, thus not all the leached cations resulted from the ion exchange process.

We cannot exclude other plausible mechanisms such as surface complexation and surface precipitation that can contribute to Pb(II) adsorption with these adsorbents (Li et al. 2017; Yang et al. 2019). As demonstrated above, these carbons are enriched with sulfur species and it is known that lead ions can interact with this type of functionalities (Saha et al. 2016), specifically with ZnS (Yan et al. 2015; Kromah and Zhang 2021).

To go deeper into the Pb(II) removal mechanism, XRD difractogramas of the CA and CA-CO2 samples after the adsorption assays were obtained (Figure S10). It was possible to observe that the XRD pattern of CA char did not change substantially, however CA-CO2 sample presented a quite different difractogram after the adsorption of Pb(II) ions. A new phase was identified, namely hydrocerussite (Pb_3_(CO_3_)_2_(OH)_2_) (COD number 9011388), indicating that removal through precipitation ocurred (Yang et al. 2019). The precipitation mechanism for Pb removal may have promoted the increase of Ca, Mg and Zn in solution due to the dissolution of Ca/Mg/Zn carbonate minerals providing carbonates for Pb precipitation (Cao et al. 2009). It was assumed that samples from rubber B presented the same behaviour.

Concerning W(VI), no studies on tungsten ion adsorption performed by chars or porous carbons were reported in the literature, apart from the work of Dias et al*.* (Dias et al. 2021). Those authors tested rice waste-derived porous carbons as highly efficient adsorbents of W(VI) oxyanions obtaining a maximum uptake capacity of 854 mg W /g activated carbon. Although the uptake capacities obtained in the present work are far away from that value, they are comparable to or even higher than those obtained with many adsorbents reported in the literature (Table S1).

The most probable mechanism ruling out W(VI) adsorption must have been strong electrostatic attractions between the negatively charged tungstate species and the highly positively charged carbons’ surface, as discussed in Sect. 3.2.1. Given the higher pH_PZC_ of the CO_2_-activated chars (Table 1), it is expected that these carbons are more positively charged at acidic pH than the raw chars, which can explain their higher attraction of W(VI) oxyanions.

## Conclusions

Spent tire rubber-derived chars and their corresponding H_3_PO_4_ and CO_2_ activated samples were applied as adsorbents in the recovery of Pb(II) ion and (W(VI)) oxyanion from synthetic solutions. The carbon samples were extensively characterized, and it was observed that the H_3_PO_4_ activated chars presented lower surface areas than the raw chars and an acidic surface chemistry which affected their performance as adsorbents of the target metal ions. It was assumed that these carbon samples presented a low content of species able to interact with the target ions. On the other hand, CO_2_-activated chars presented increased surface areas and increased mineral content compared to the raw chars, which conferred them higher uptake capacities for both Pb(II) and W(VI) ions. Their better performance was attributed mainly to an increase in the content of exchangeable cations. Also, the CO_2_ samples presented the higher kinetic constants of the adsorption process.

Cation exchange with Ca, Mg and Zn ions was appointed as a mechanism for Pb removal, as well surface precipitation in the form of hydrocerussite (Pb_3_(CO_3_)_2_(OH)_2_). W(VI) adsorption might have been ruled by strong electrostatic attractions between the negatively charged tungstate species and the highly positively charged carbons’ surface.

The results presented in this work allow concluding that the valorization of spent tire rubber through pyrolysis and the subsequent activation of the obtained chars is an alter-native option to obtain efficient adsorbents for the removal and recovery of critical metallic elements.

## Supplementary Information

Below is the link to the electronic supplementary material.

Supplementary file1 (PDF 1.24 MB)

## Data Availability

Data and materials can be available upon request to the authors.
