# Supplementary material for: Valorisation of spent tire rubber as carbon adsorbents for Pb(II) and W(VI) in the framework of a Circular Economy
Source: Environ Sci Pollut Res Int. 2023 May 20;30(30):74820–37. doi: 10.1007/s11356-023-27689-5 (PMC10293437; doi:10.1007/s11356-023-27689-5)
Supplement: Supplementary file 1 — Supplementary file1 (PDF 1.24 MB) [file 11356_2023_27689_MOESM1_ESM.pdf]

Supplementary material

Valorization of spent tire rubber as carbon adsorbents for Pb(II) and W(VI) in the framework of a Circular Economy

Maria Bernardo[1*], Nuno Lapa[1], Filomena Pinto[2], Miguel Nogueira[1], Inês Matos[1], Márcia Ventura[1], Ana Maria Ferraria[3,4], Ana Maria Botelho do Rego[3,4], Isabel Maria Fonseca[1]

*Corresponding author:

Maria Bernardo (Tel.: +351 212948300; email: maria.b@fct.unl.pt)

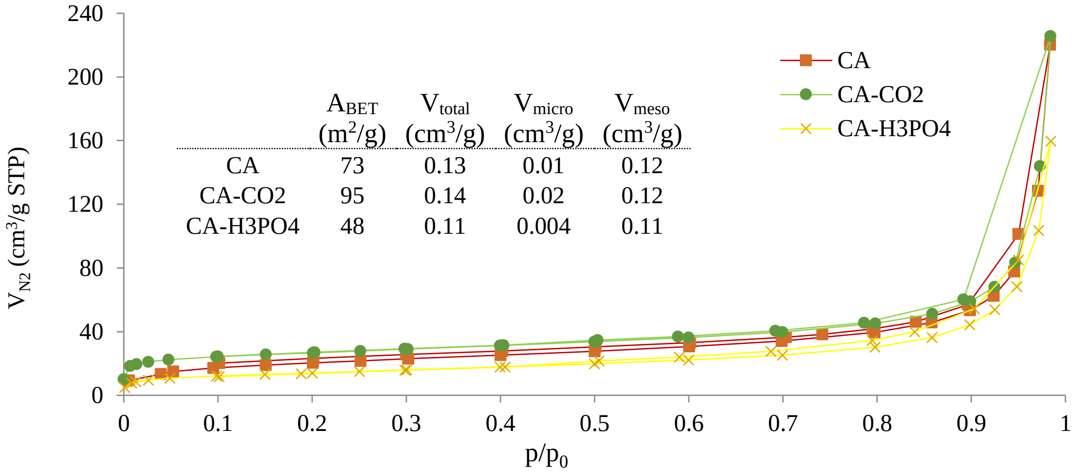

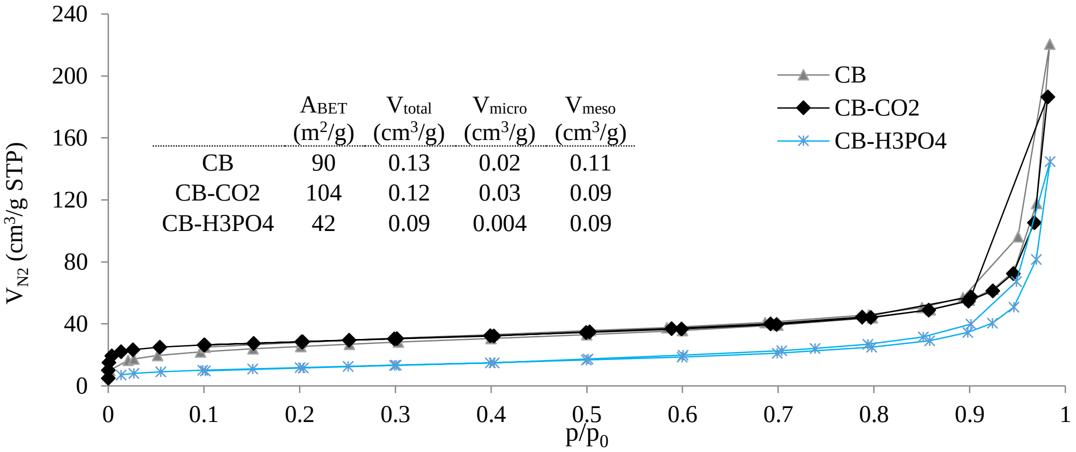

Fig. S1 N_2 adsorption-desorption isotherms of rubber A (above) and rubber B (below) derived carbons

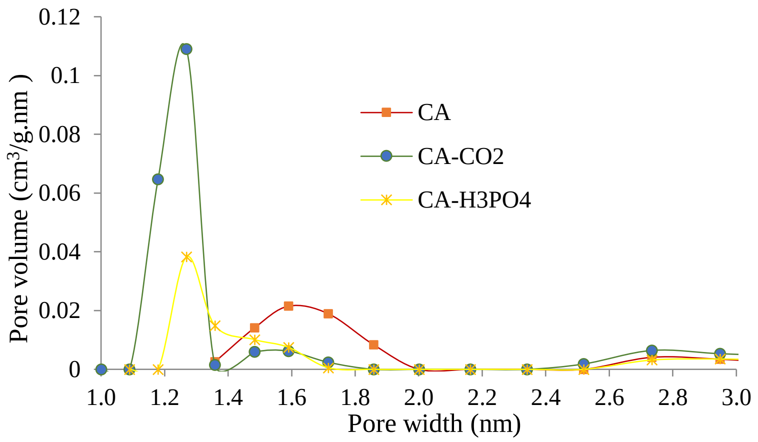

Fig. S2 Micropore size distribution from DFT adsorption model for carbon slit-shaped pores of rubber A-derived carbons

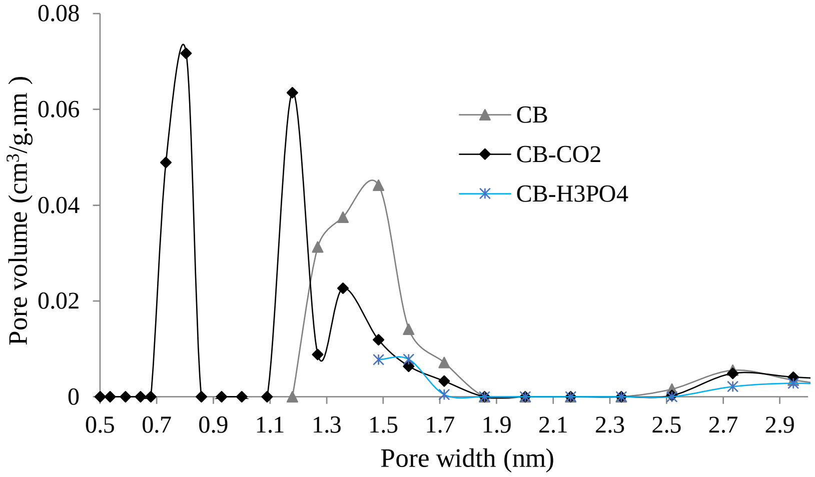

Fig. S3 Micropore size distribution from DFT adsorption model for carbon slit-shaped pores of rubber B-derived carbons

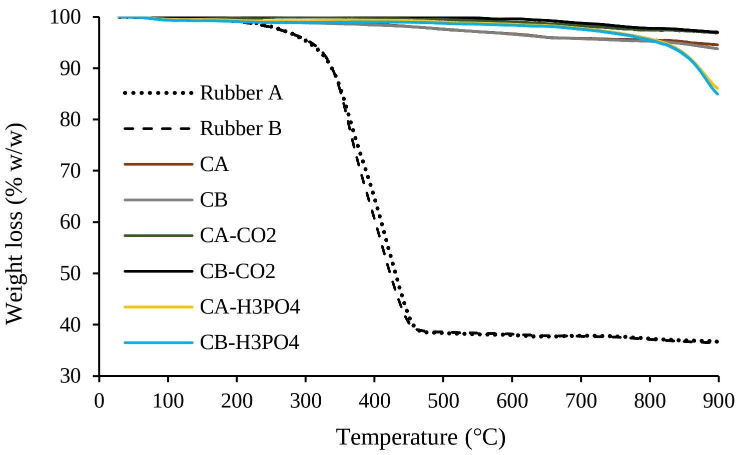

Fig. S4 TGA curves for spent tire rubbers and carbon samples

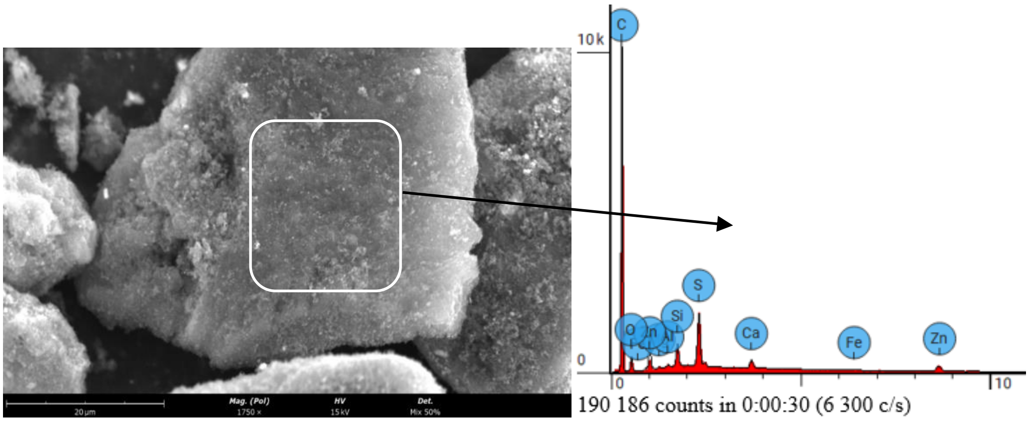

Fig. S5 SEM image of a CA-CO2 particle with 1750× magnification and the corresponding EDS spectrum

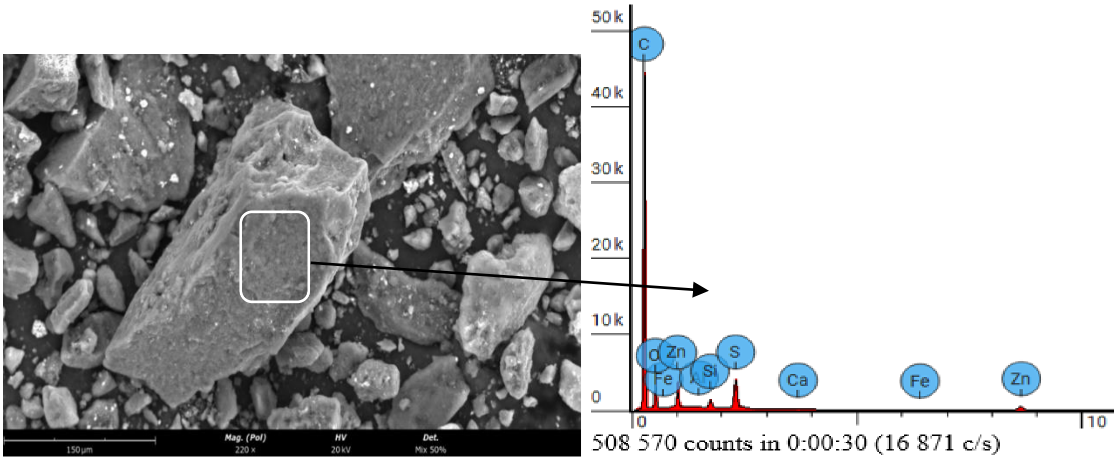

Fig. S6 SEM image of CB-CO2 particles with 220× magnification and the corresponding EDS spectrum

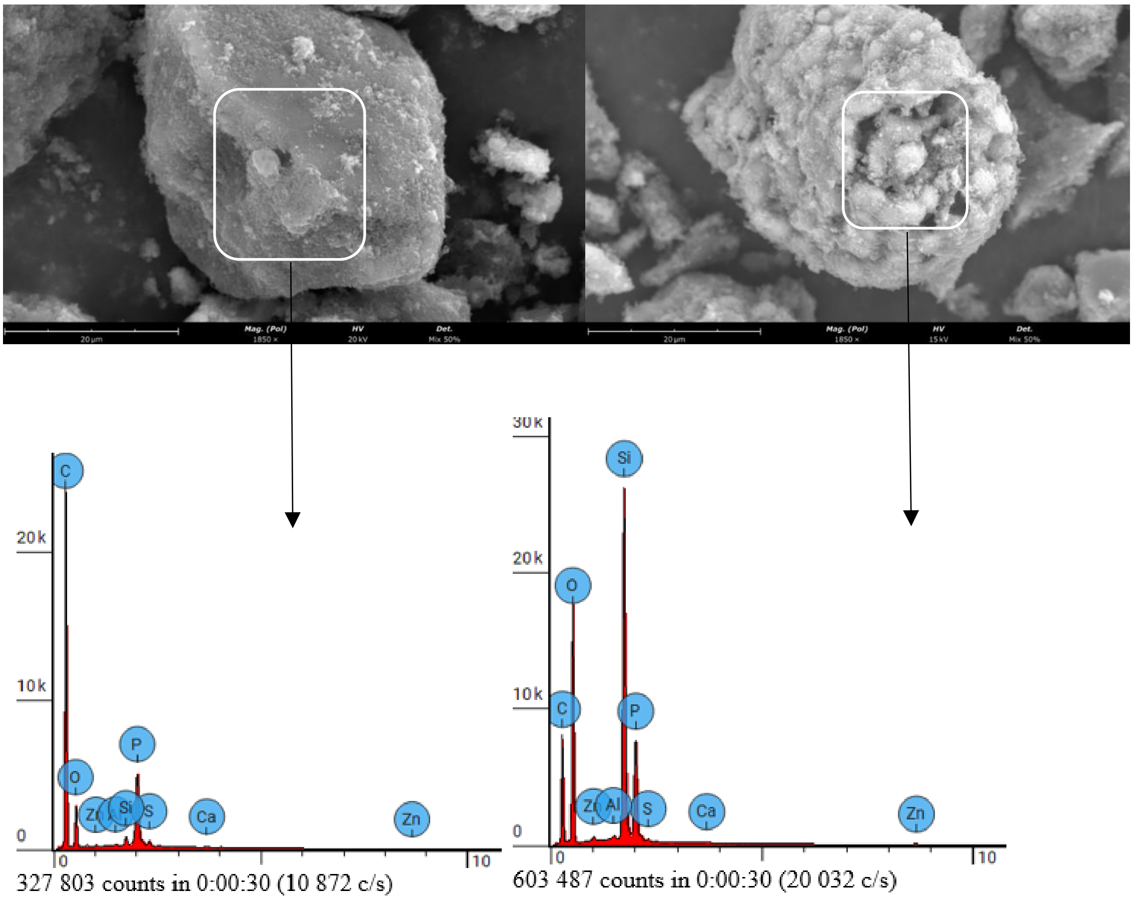

Fig. S7 SEM images of CA-H3PO4 particles with 1850× magnification (both images) and the corresponding EDS spectra

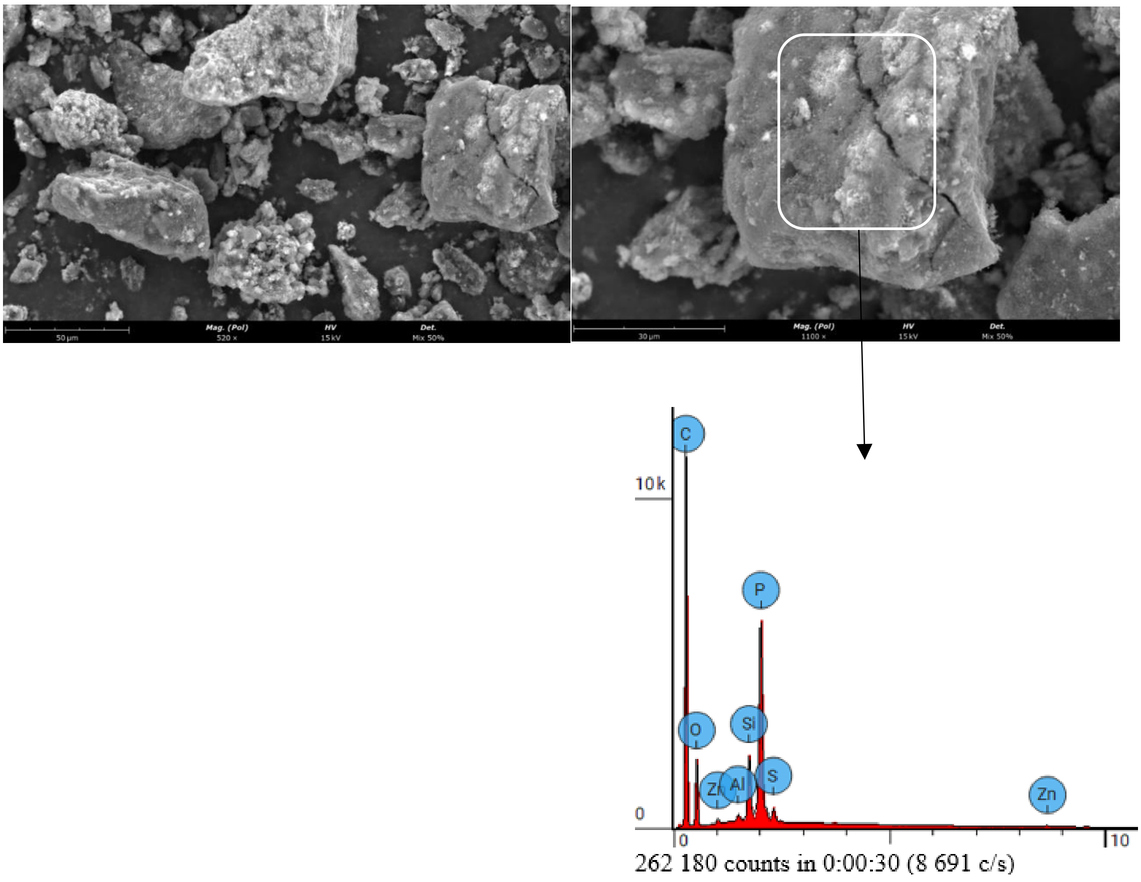

Fig. S8 SEM images of CB-H3PO4 particles with 520x (left) and 1100× (right) magnification and the corresponding EDS spectrum

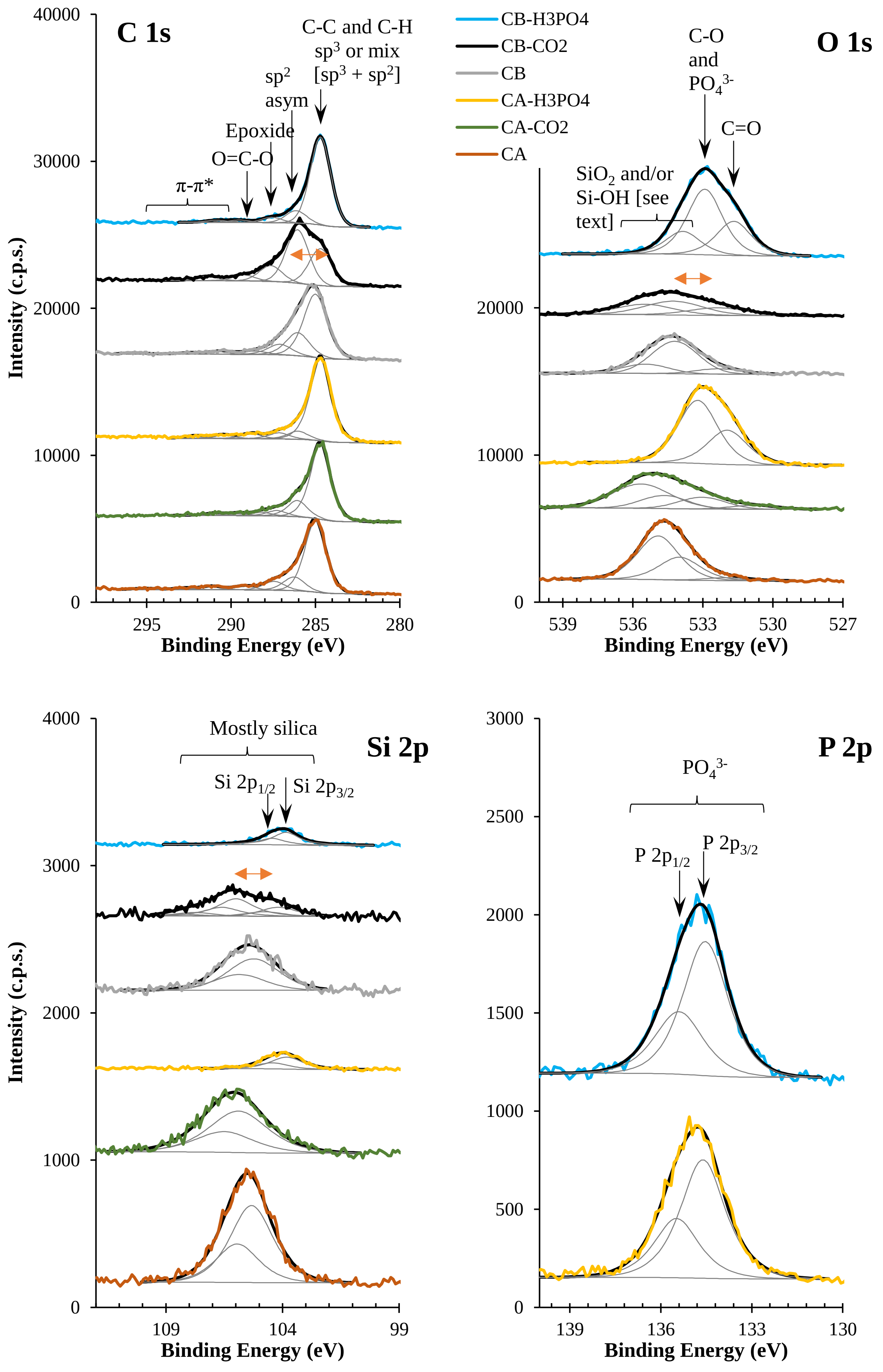
C 1s
C-C and C-H
sp3 or mix
[sp3 + sp2]
sp2
asym
Epoxide
O=C-O
π-π*
CB-H3PO4
CB-CO2
CB
CA-H3PO4
CA-CO2
CA
O 1s
C-O
and
PO4 3-
C=O
SiO2 and/or
Si-OH [see
text]
Si 2p
Mostly silica
Si 2p1/2
Si 2p3/2
P 2p
PO4 3-
P 2p1/2
P 2p3/2
Intensity (c.p.s.)
Binding Energy (eV)

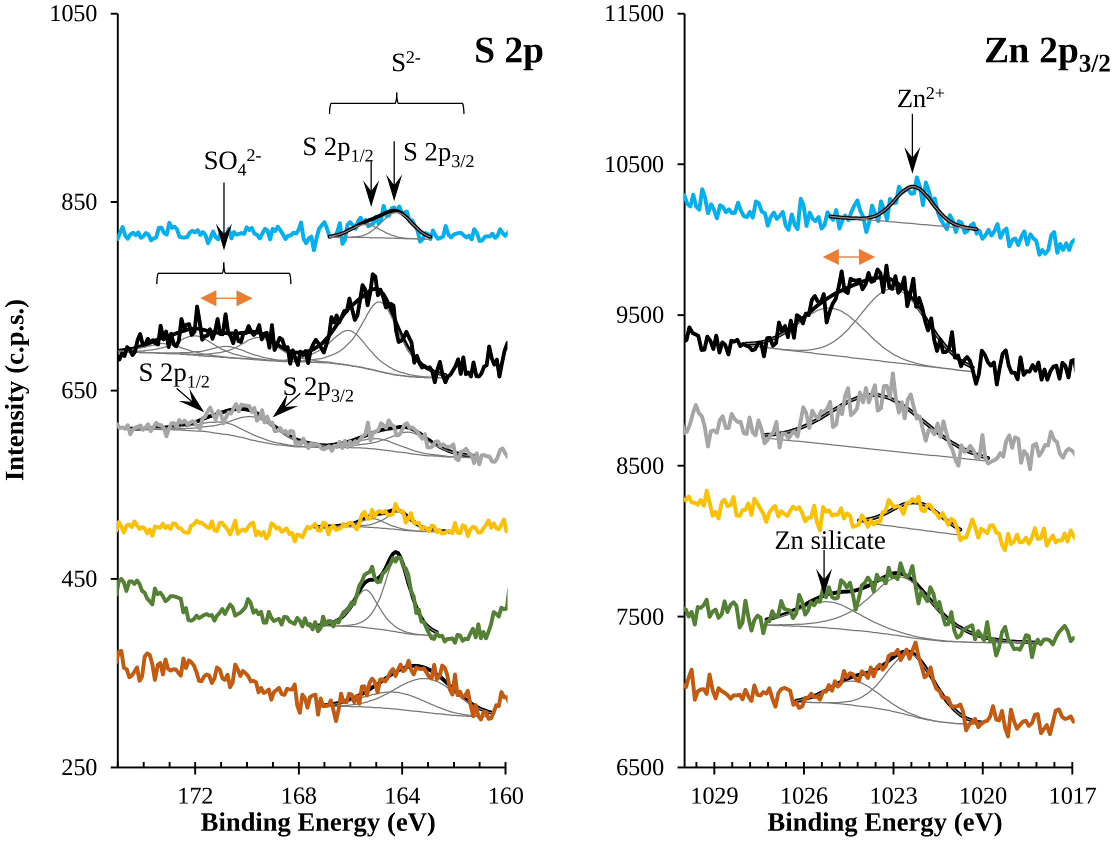

Fig. S9 XPS regions C 1s, O 1s, Si 2p, P 2p, S 2p and Zn 2p3/2. The color code is the same for all regions (From bottom to top: CA, CA-CO2, CA-H3PO4, CB, CB-CO2, CB-H3PO4)

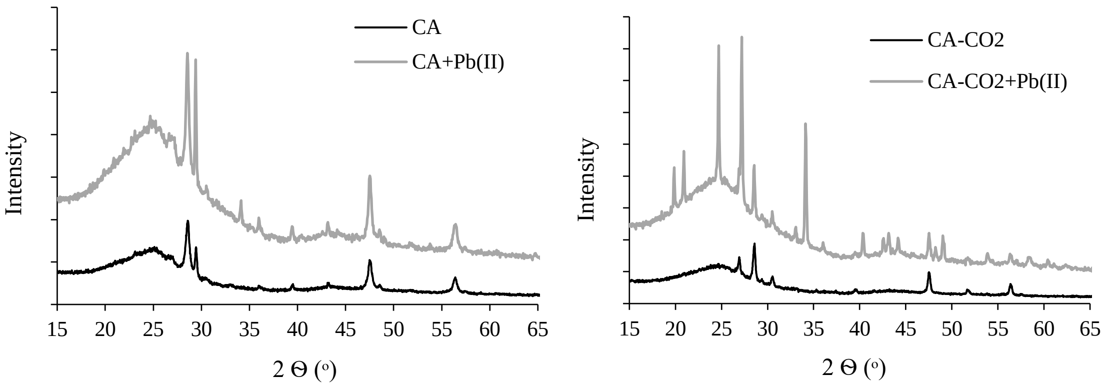

Fig. S10 XRDP pattern of activated chars from rubber A before and after adsorption of Pb(II) ions

Table S1. Comparison of the uptake capacities of Pb(II) and W(VI) obtained in the present work with the ones presented in the literature.

Adsorbent	Activation/ Treatment	Pb (II) uptake capacity (mg/g)	W (VI) uptake capacity (mg/g)	Reference
Tire-derived char	O_2, CO_2 and steam activation	~ 20.0	-	(Helleur et al. 2001)
Tire-derived char	Impregnation with $Fe(OH)_3$	4.97	-	(Ramola et al. 2014)
Tire-derived char	Steam activation	24.0	-	(Lu et al. 2016)
Tire-derived char	KOH activation	85.9	-	(Malise et al. 2020)
Tire-derived activated chars	CO_2 activation	103.0 – 116.0	27.1 – 30.7	This work
Hydrotalcite	-	-	50.0 – 59.5	(Ogata et al. 2017)
Multi-Walled Carbon Nanotubes	-	-	20.6	(Ihsanullah et al. 2020)
Zeolite fly-ash	-	-	33.4	(Ogata et al. 2014)
Iowaite	-	-	54.1	(Cao et al. 2019)
Sepiolite	H_2SO_4 treatment	-	35.2	(Wang et al. 2015)
Pyrite	-	-	4.65 – 13.2	

References

Cao Y, Guo Q, Shu Z, et al (2019) Tungstate removal from aqueous solution by nanocrystalline iowaite: An iron-bearing layered double hydroxide. Environ Pollut 247:118–127. doi: 10.1016/j.envpol.2019.01.021

Helleur R, Popovic N, Ikura M, et al (2001) Characterization and potential applications of pyrolytic char from ablative pyrolysis of used tires. J Anal Appl Pyrolysis 58–59:813–824. doi: 10.1016/S0165-2370(00)00207-2

Ihsanullah I, Sajid M, Kabeer M, et al (2020) First Investigations on the Removal of Tungsten Species from Water Using Multi-walled Carbon Nanotubes. Water, Air, Soil Pollut 231:119. doi: 10.1007/s11270-020-04485-2

Lu P, Shen T-Y, Zhang C (2016) Adsorption of Pb(II) by Activated Pyrolytic Char from Used Tire. MATEC Web Conf 67:01009. doi: 10.1051/matecconf/20166701009

Malise L, Rutto H, Seodigeng T, et al (2020) Adsorption of Lead Ions onto Chemical Activated Carbon Derived From Waste Tire Pyrolysis Char: Equilibrium and Kinetics Studies. Chem Eng Trans. doi: 10.3303/CET2082071

Ogata F, Iwata Y, Kawasaki N (2014) Adsorption of Tungsten onto Zeolite Fly Ash Produced by Hydrothermally Treating Fly Ash in Alkaline Solution. Chem Pharm Bull 62:892–897. doi: 10.1248/cpb.c14-00291

Ogata F, Nakamura T, Ueta E, et al (2017) Adsorption of tungsten ion with a novel Fe-Mg type hydrotalcite prepared at different Mg2+/Fe3+ ratios. J Environ Chem Eng 5:3083–3090. doi: 10.1016/j.jece.2017.06.017

Ramola S, Mishra T, Rana G, Srivastava RK (2014) Characterization and pollutant removal efficiency of biochar derived from baggase, bamboo and tyre. Environ Monit Assess 186:9023–9039. doi: 10.1007/s10661-014-4062-5

Wang Y, Chen K, Mo L, et al (2015) Removal of tungsten from electroplating wastewater by acid- and heat-treated sepiolite. Desalin Water Treat 56:232–238. doi: 10.1080/19443994.2014.934724
